# Soy-Induced Fecal Metabolome Changes in Ovariectomized and Intact Female Rats: Relationship with Cardiometabolic Health

**DOI:** 10.1038/s41598-018-35171-3

**Published:** 2018-11-15

**Authors:** Victoria J. Vieira-Potter, Tzu-Wen L. Cross, Kelly S. Swanson, Saurav J. Sarma, Zhentian Lei, Lloyd W. Sumner, Cheryl S. Rosenfeld

**Affiliations:** 1 Department of Nutrition and Exercise Physiology, University of Missouri, Columbia, MO 65211 USA; 2 Department of Bacteriology, University of Wisconsin-Madison, Madison, WI 53706 USA; 3 Division of Nutritional Sciences, University of Illinois at Urbana-Champaign, Urbana, IL 61801 USA; 4 Department of Animal Sciences, University of Illinois at Urbana-Champaign, Urbana, IL 61801 USA; 5 MU Metabolomics Center, University of Missouri, Columbia, MO 65211 USA; 6 Biochemistry, University of Missouri, Columbia, MO 65211 USA; 7 Bond Life Sciences Center, University of Missouri, Columbia, MO 65211 USA; 8 Biomedical Sciences, University of Missouri, Columbia, MO 65211 USA; 9 Thompson Center for Autism and Neurobehavioral Disorders, University of Missouri, Columbia, MO 65211 USA; 10 Genetics Area Program, University of Missouri, Columbia, MO 65211 USA

## Abstract

Phytoestrogens are plant-derived compounds found in a variety of foods, most notably, soy. These compounds have been shown to improve immuno-metabolic health, yet mechanisms remain uncertain. We demonstrated previously that dietary phytoestrogen-rich soy (SOY) rescued metabolic dysfunction/inflammation following ovariectomy (OVX) in female rats; we also noted remarkable shifts in gut microbiota in SOY vs control diet-fed rats. Importantly, specific bacteria that significantly increased in those fed the SOY correlated positively with several favorable host metabolic parameters. One mechanism by which gut microbes might lead to such host effects is through production of bacterial metabolites. To test this possibility, we utilized non-targeted gas chromatography–mass spectrometry (GCMS) to assess the fecal metabolome in those previously studied animals. Partial least square discriminant analysis (PLSDA) revealed clear separation of fecal metabolomes based on diet and ovarian state. In particular, SOY-fed animals had greater fecal concentrations of the beneficial bacterial metabolite, S-equol, which was positively associated with several of the bacteria upregulated in the SOY group. S-equol was inversely correlated with important indicators of metabolic dysfunction and inflammation, suggesting that this metabolite might be a key mediator between SOY and gut microbiome-positive host health outcomes.

## Introduction

Phytoestrogens from dietary soy protein have estrogen-like selective estrogen receptor modulator (SERM) effects. This dietary approach holds promise as an alternative therapeutic approach to estrogen for reducing adiposity and improving insulin resistance^1,2^. Although substantial evidence supports the cardio-metabolic benefits of phytoestrogens^3–11^, the underlying mechanisms remain poorly understood. Our data in ovariectomized (OVX) and non-ovariectomized (SHM) rats reveal that a soy-based diet with known phytoestrogen concentrations (i.e. SOY) improves metabolism via a mechanism independent of either energy consumption or physical activity^12^. The most notable changes induced by SOY were those associated with gut microbial population shifts^12^. Further, significant relationships between important immuno-metabolic outcomes (e.g., adipose tissue inflammation) and specific microbes induced by SOY were identified.

Other animal model and human studies have shown that soy-based diets and phytoestrogen supplementation alone can result in positive gut microbiome changes^13–22^. One possible mechanism by which the gut microbiome might affect host function is through production of unique bacterial metabolites^23–28^. One category of bacterial metabolites that are increased in animals and humans consuming a soy-rich diet and that might have important health benefits are the equols, in particular S-equol^29–31^. Metabolism of phytoestrogens by gut microbes may produce metabolites with more potent biological activity within the host than their parental compounds^32^. Equols are metabolites formed by reduction of soy isoflavones by gut microorganisms^33,34^. S-equol is an important metabolite, particularly among postmenopausal women, because it has estrogenic effects with greater binding affinity for estrogen receptors than isoflavones in their premetabolized form^35^. Interestingly, there is considerable inter-individual variation in the metabolism of isoflavones to equol, and dietary factors (e.g., high fat diet may reduce equol production)^36^ also have been shown to affect its production. While S-equol represents one key bacterial metabolite induced by consumption of a soy diet, there are presumably others yet to be identified that may also affect host metabolic responses. However, S-equol has been the most well-studied metabolite in this regard, and many lines of evidence support that it has beneficial metabolic and behavioral effects. Moreover, other constituents present in whole soy protein (e.g., oligosaccharides) may affect gut microbial metabolism^37^ in such a way as to increase bacterial phytoestrogen metabolism. Thus, it is important to investigate the effects of whole soy on gut microbial metabolism.

The goal of the current study was to examine the fecal metabolome in the groups of SHM and OVX rats previously demonstrated to exhibit SOY-mediated improvements in a variety of immuno-metabolic improvements, along with gut microbiota changes that associated with those imporved metabolic outcomes^12^. We also examined whether ovarian state interacted with diet to affect the fecal metabolome. The metabolites identified to be altered by SOY were then correlated with the previously identified gut microbes upregulated in the SOY-fed rats. Finally, we performed correlation analyses to determine how the bacterial metabolites increased in the SOY group associated with previously measured physiological parameters and gene expression patterns in white adipose tissue (WAT) and brown adipose tissue (BAT).

## Results

### Soy-Induced Fecal Metabolome Differences

**Figure 1:**
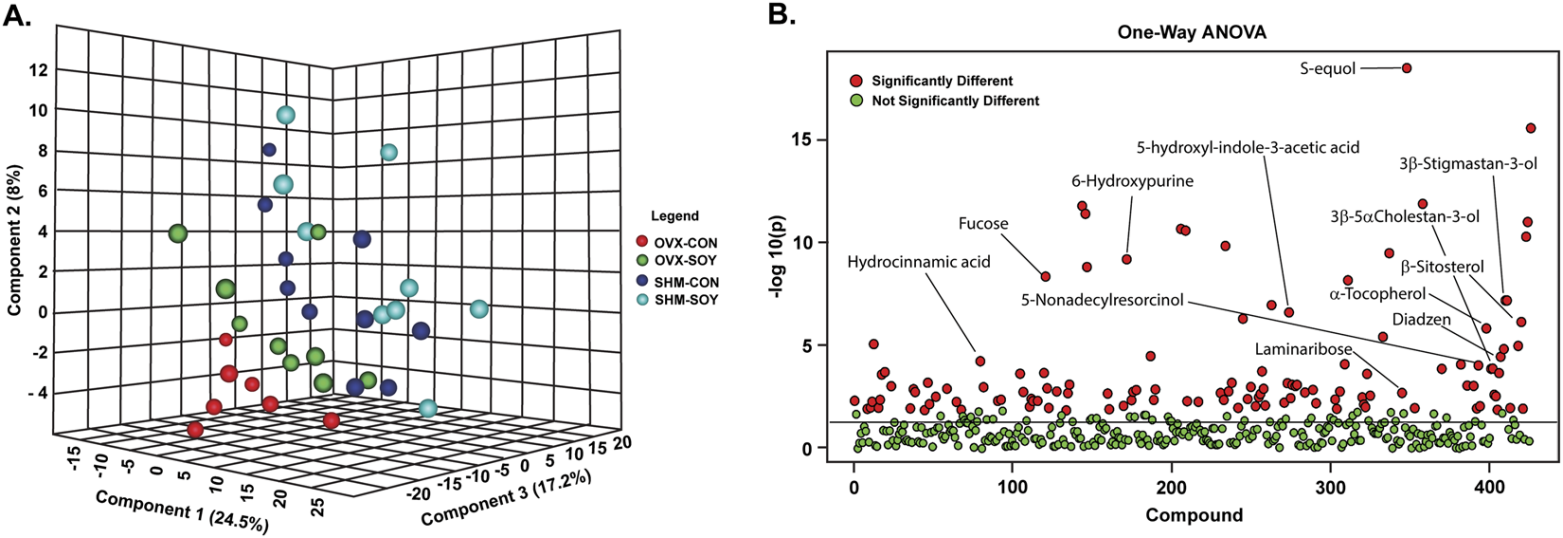
General characterization of fecal metabolome data from SOY and CON fed OVX and SHM female rats. (**A**) 3D score plot of Partial Least Square-Discriminant Analysis considering ovarian state and diet both as variants. (**B**) One-way ANOVA with Fisher’s LSD post-hoc analysis revealing the statistical differences in metabolite changes considering ovarian state and diet both as variants. Y-axis represents the log10 value of *p* value with a horizontal line at *p* = 0.05. Those that did not differ significantly are shown in green, and those that were significantly different in at least one of the groups are indicated in red. *p* < 0.05.

The metabolomics data preprocessing was performed using the R programming language (https://www.r-project.org/). The samples were then analyzed with PLS-DA (Partial Least Squares Discriminant Analysis), which revealed clear separation based on diet (SOY vs. CON) and ovarian state (OVX vs. SHM) and was significantly different based on confidence testing (*p* = 0.048) (Fig. 1A). Differential clustering was confirmed by analyzing outliers in t-test/volcano plots or ANOVA (in case of four groups). One-way ANOVA was then used to determine the overall number of metabolites that differed based on diet and ovarian state (Fig. 1B). Additionally, two-way ANOVA was used to confirm main effects of diet and ovarian state and the interaction of diet by ovarian state (Supplementary Table 1).

**Figure 2:**
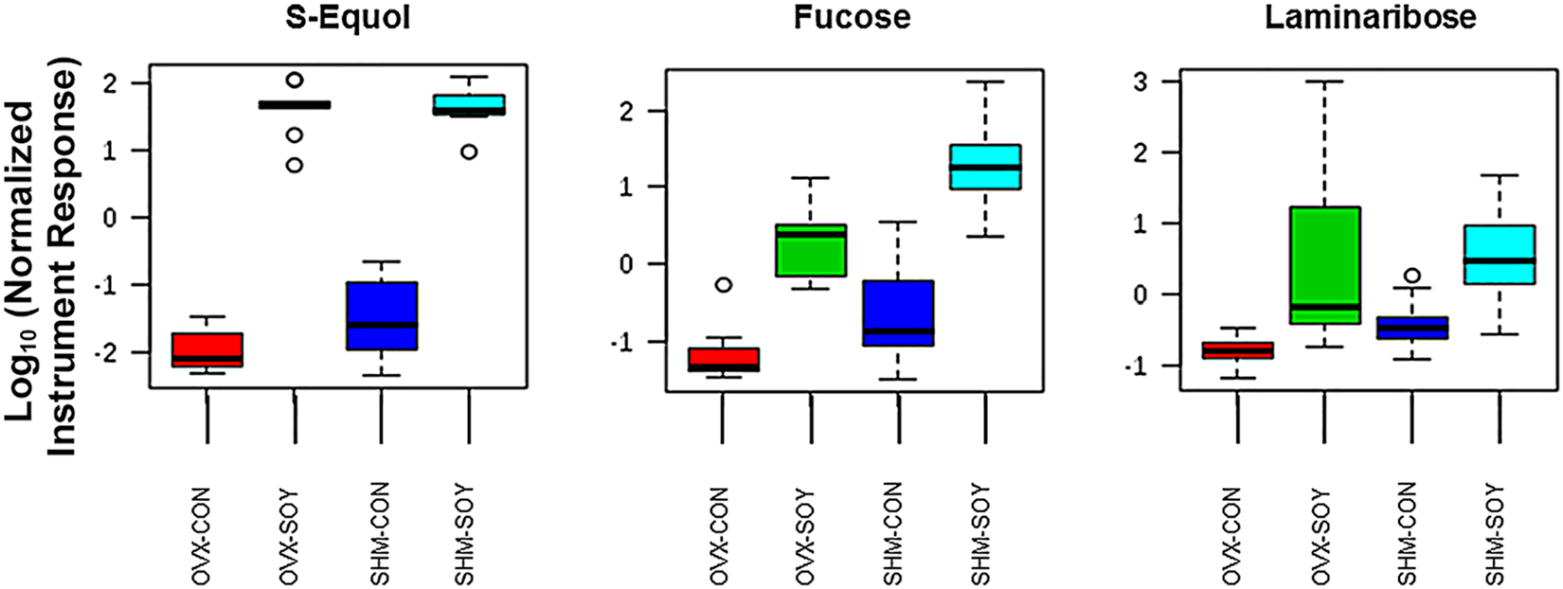
Traditional box plots for metabolites elevated in SOY vs. CON females for both OVX and SHM groups, *p* < 0.05. The Y axes are log 10 values of the normalized instrument response for the labeled metabolites (x-axes). The program arbitrarily assigns color codes for the various groups. The box plot upper and lower brackets represents +/− (1.58*interquartile range-IQR/Squared root of sample size). To reduce ambiguity, the one-way ANOVA comparisons as determined by the MetaboAnalyst software program included OVX-CON vs. OVX-SOY and SHM-CON vs. SHM-SOY. A complete list of metabolites that differed between these two groups and directionality is included in Supplementary File 1.

**Figure 3:**
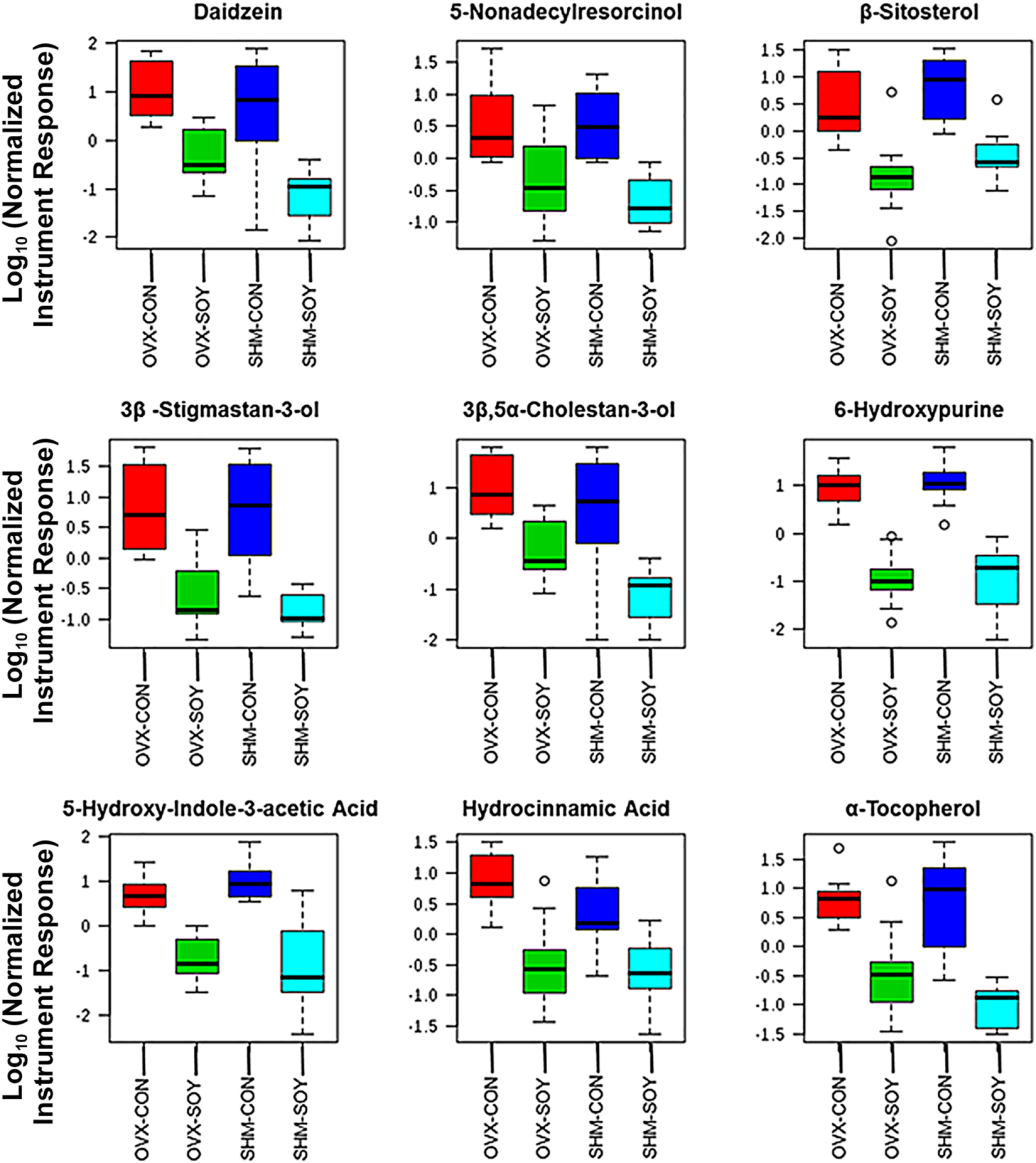
Traditional box plots for metabolites decreased in SOY vs. CON females for both OVX and SHM groups, p < 0.05. The Y axes are log 10 values of the normalized instrument response for the labeled metabolites (x-axes). The program arbitrarily assigns color codes for the various groups. The box plot upper and lower brackets represents +/− (1.58*IQR/Squared root of sample size). To reduce ambiguity, the one-way ANOVA comparisons as determined by the MetaboAnalyst software program included OVX-CON vs. OVX-SOY and SHM-CON vs. SHM-SOY. A complete list of metabolites that differed between these two groups and directionality is included in Supplementary File 1.

Examples of elevated metabolites in SOY vs. CON regardless of ovarian state are shown in Fig. 2. Notably, S-equol was one of the top metabolites that was significantly increased in both groups of SOY-fed rats (Fig. 2A). Other metabolites that were increased in the SOY groups include fucose, laminaribose, and several currently uncharacterized metabolites. We also examined the metabolites that were decreased, suggestive of bacterial consumption or metabolism, in SOY vs. CON groups, and these include daidzein; 5-nonadecylresorcinol; β-sitosterol; 3β-stigmastan-3-ol; 3β, 5α-cholestan-3-ol; 6-hydroxypurine; 5-hydroxy-indole-3-acetic acid; hydrocinnamic acid; and α-tocopherol (Fig. 3). The full list of fecal metabolites that increased or decreased in SOY-fed individuals is provided in Supplementary File 1, those highlighted in orange are significantly different.

### Metabolic Pathways Predicted to be Affected in SOY groups

Based on the overall metabolite differences in the SOY vs. CON groups, two pathways are predicted to be affected in the SOY groups: (1) glycine, serine, and threonine metabolism and (2) aminoacyl-tRNA biosynthesis (q values, false discovery rate-FDR, =0.0004 and 0.1, respectively, Supplementary File 2). Metabolome view of pathway analysis was done using Metaboanalyst software, and the four most significant pathways are labeled (Supplementary Fig. 1). As shown in the KEGG pathway map for glycine, serine, and threonine metabolism^38–40^, some of the individual metabolites in this pathway were increased; whereas others were decreased (Supplementary Fig. 2).

### Interactive Effects of Diet and Ovarian State on Fecal Metabolome

To examine whether ovarian state (OVX or SHM) within each diet might affect the fecal metabolome, we examined the metabolite differences in OVX vs. SHM for those fed the SOY diet (Supplementary Figs 3 and 4) and those fed the CON diet (Supplementary Figs 5 and 6). Supplementary Fig. 3 shows example metabolites that were increased in OVX compared to SHM individuals within the SOY diet, such as phenylpyruvic acid; 3β, 5β-cholestan-3-ol, 3β, 5α-cholestan-3-ol; 1-octadecane; 24-ethyl-δ(22)-coprostenol; and L-Alanine. In Supplementary Fig. 4, example metabolites decreased in OVX compared to SHM individuals within the SOY diet are shown. These include fucose; rhamnose; D-(+)Talose; and galacturonic acid.

Comparison of bacterial metabolites that differed in OVX vs. SHM individuals fed the CON diet, reveals that L-serine; L-valine; 2-oxobutanoic acid; hexanoic acid; α-ketovaleric acid; and 2-oxo-isocaproic acid are example metabolites that were increased in OVX compared to SHM females (Supplementary Fig. 5). In contrast, pipecolic acid and 5-aminovaleric acid were select metabolites that decreased in OVX compared to SHM individuals when fed the CON diet (Supplementary Fig. 6).

### Correlation of SOY Effects on the Fecal Metabolome and Gut Microbiota

**Figure 4:**
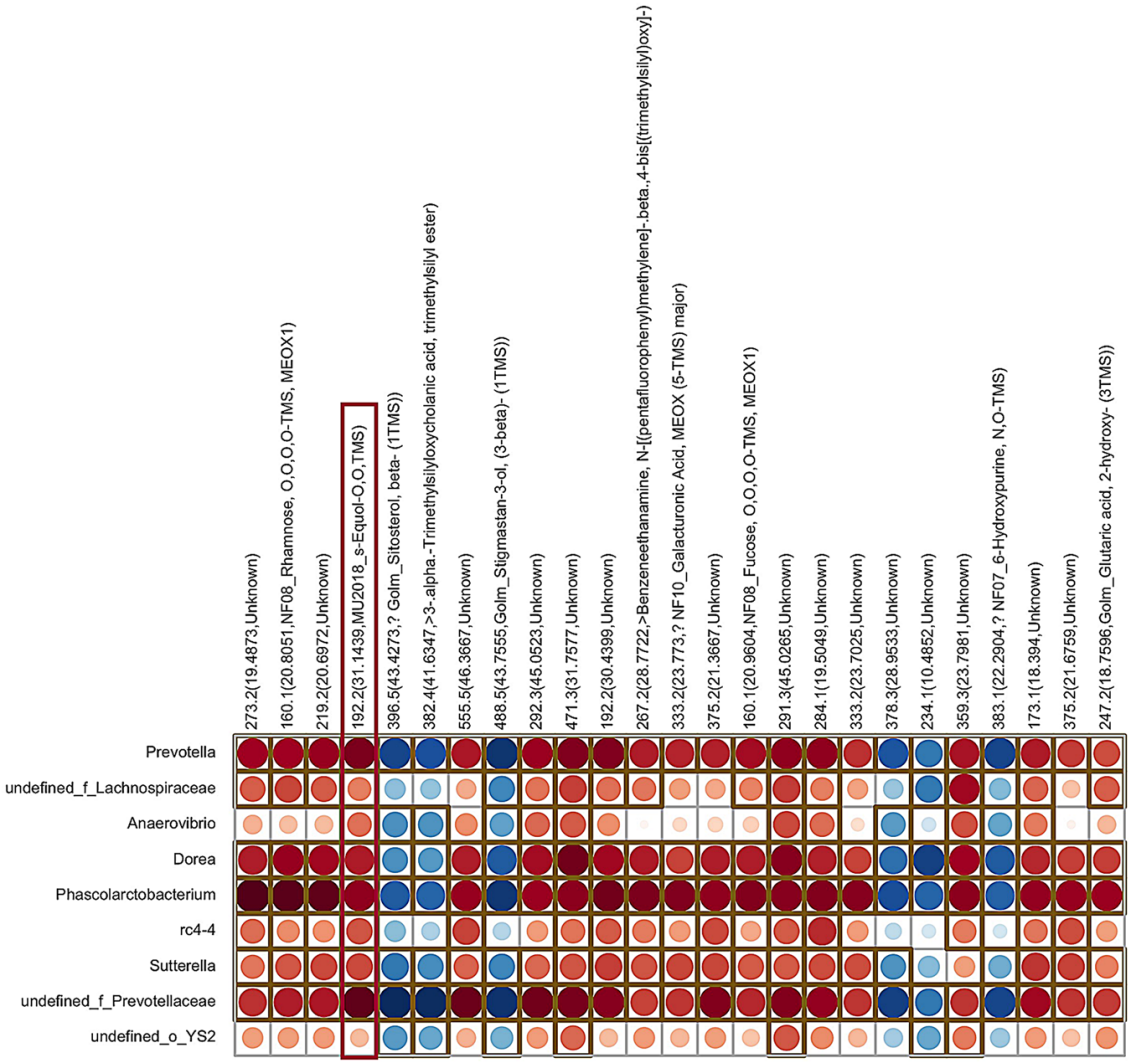
Correlations among taxa increased in cecal microbial community of SOY-fed rats and fecal metabolomic changes due to SOY diet consumption. One metabolite that strongly correlated with relative elevations in select bacteria was S-equol (boxed in region). The shading intensity of the bubble, along with size, is indicative of the Spearman rank correlation coefficient between variables. Red dots represent positive correlations whereas blue dots represent negative correlations; brown square box denotes statistical significance (p < 0.05) observed using Spearman correlation; N = 35 total animals.

**Figure 5:**
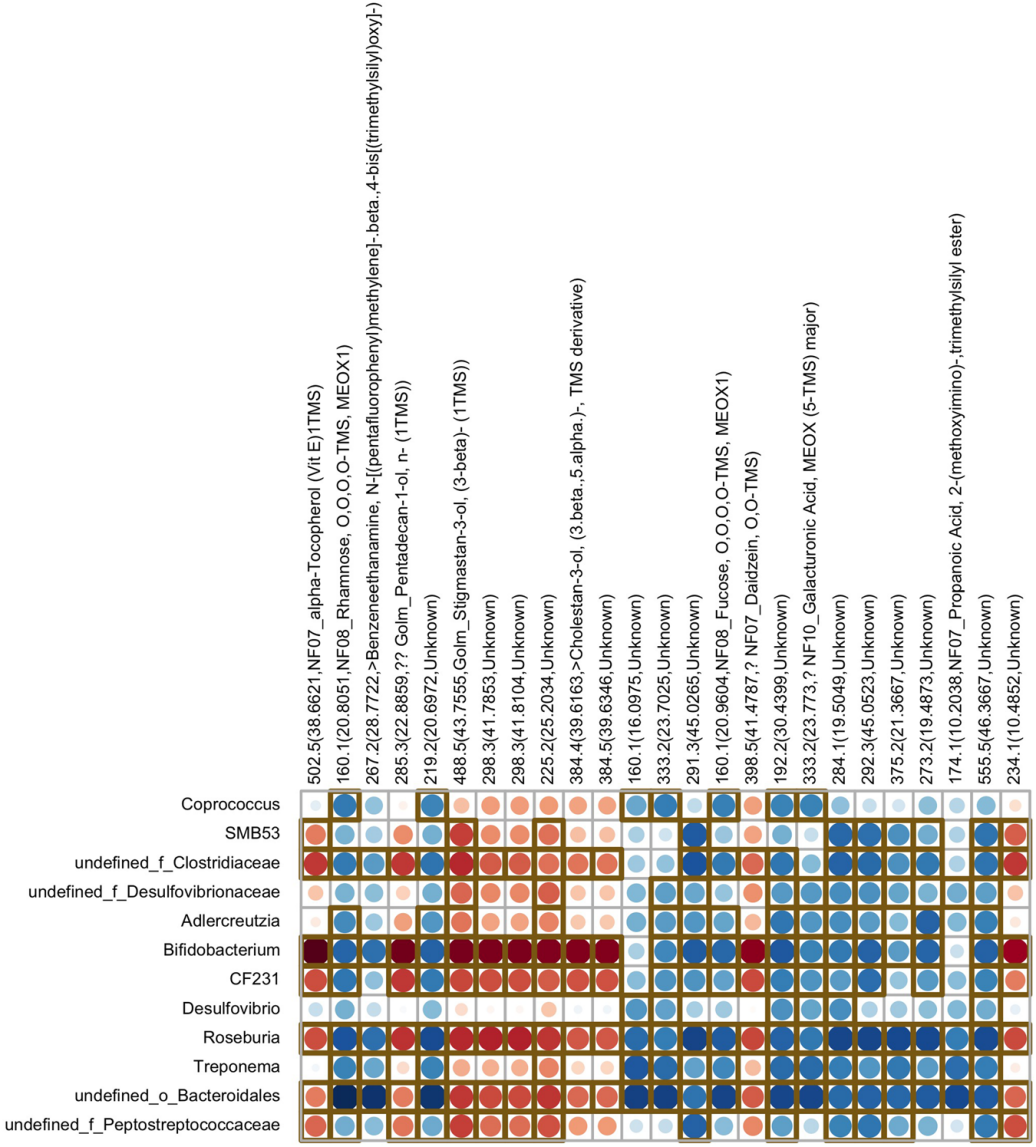
Correlations among taxa decreased in cecal microbial community of SOY-fed rats and fecal metabolomic changes due to SOY diet consumption. The shading intensity of the bubble, along with size, is indicative of the Spearman rank correlation coefficient between variables. Red dots represent positive correlations whereas blue dots represent negative correlations; brown square box denotes statistical significance (p < 0.05) observed using Spearman correlation; N = 35 total animals.

To determine whether selected bacteria affected by the SOY diet might be linked, and possibly account for, the increased or decreased fecal metabolites detailed above, we performed correlation analyses with cecal bacteria increased in SOY groups (Fig. 4) and those that were decreased in the SOY groups^12^ (Fig. 5) and correlated them with the top 25 fecal metabolites identified to be different based on *p* value association. As shown in Fig. 4, a relative increase in *Prevotella spp*., *Dorea spp*., *Sutterella spp*., and *Phascolarctobacterium* was positively associated with an increase in S-equol, rhamnose, benzeneethanamine, galacturonic acid, fucose, and several currently unknown metabolites. Conversely, increases in these cecal bacteria were associated with decreases in fecal stigmastan-3-ol and 6-hydroxypurine. Correlation of the bacteria that were decreased in the SOY groups and the top 25 metabolite changes revealed that an undefined genus within the family Clostridiaceae, *Bifidobacterium spp*., CF231, *Rosebura spp*., and an undefined genus within the family Bacteroidales were associated with similar metabolite changes (Fig. 5). Relative decreases in bacteria were associated with reductions in α-tocopherol, pentadecan-1-ol, stigmastan-3-ol, cholestan-3-ol, and daidzein. In contrast, relative declines in those bacteria were associated with increases in rhamnose, benzeneethanamine, fucose, galacturonic acid, propanoic acid, and several currently uncharacterized metabolites.

### Correlations Between Fecal Metabolites, Physiological Parameters, and Gene Expression Results

**Figure 6:**
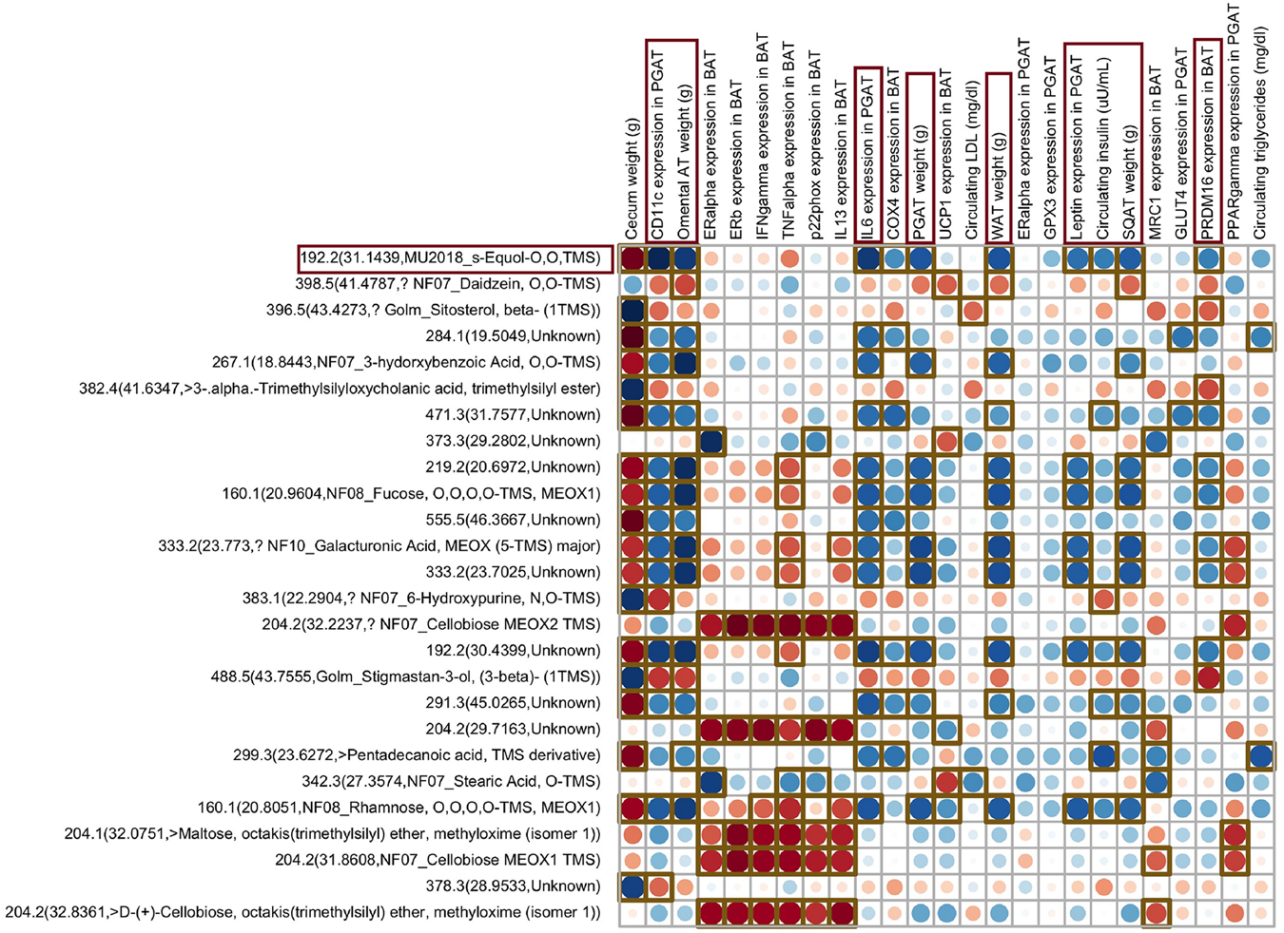
Correlations between the fecal metabolites and physiological parameters and gene expression data. As this figure shows, S-equol, which was elevated only in the SOY groups, strongly correlated with several improved metabolic outcomes (boxed in regions). Red dots represent positive correlations whereas blue dots represent negative correlations; brown square box denotes statistical significance (p < 0.05) observed using Spearman correlation; figure cropped to highlight specific correlations; N = 35 total animals.

Correlation analyses were performed to determine if fecal metabolite changes in the SOY groups correlated with previously measured physiological parameters and gene expression data in WAT and BAT^12^. S-equol was significantly increased in the SOY groups (Fig. 6 and Supplementary File 1), and greater level of this metabolite was strongly correlated with overall reduced adiposity, including decreases in omental adipose tissue (AT) and perigonadal AT (PGAT) (i.e. visceral fat depots), subcutaneous AT (SQAT), and overall adiposity. Importantly, this metabolite was also linked with reductions in inflammatory marker gene expression within AT, leptin expression in PGAT, and circulating insulin. Daidzein, a precursor of S-equol, was positively associated with omental AT, SQAT, and overall WAT weight, along with uncoupling protein 1 (*Ucp1)* and PR domain containing 16 (*Prdm16*) expression in brown adipose tissue (BAT). The collective findings suggest that the beneficial metabolic effects of daidzein may be contingent on it being metabolized to S-equol.

Fucose was also increased in the SOY groups, and this metabolite was associated with select positive metabolic outcomes, such as reduced adiposity and inflammatory marker expression in AT. An increase in cellobiose was strongly and positively associated with expression of several genes in BAT, including *Esr1*and *Esr2* (i.e., estrogen receptors), interferon-γ (*Ifng)* and tumor necrosis factor-α (*Tnfa)* (i.e., inflammatory cytokines), cytochrome B-245 Alpha Chain *(P22phox)* (i.e., oxidative stress inducer) and interleukin 13 (*Il13*) (i.e., Th2 cytokine) suggesting that this metabolite might specifically affect BAT, but interestingly, not classical WAT.

## Discussion

The overarching goal of the current study was to determine if consumption of a diet rich in soy phytoestrogens (i.e., “SOY”) by ovariectomized (OVX) and sham-operated, ovary-intact (SHM) female rats could result in fecal metabolome changes and whether such collective changes might allow for predictions to be made on which metabolic pathways might be affected in the SOY groups. We also sought to determine if ovarian state, along with diet, might affect the fecal metabolome profile. Correlation analyses were performed to determine if previously identified gut microbiota changes^12^ might account for alterations in metabolites observed in the SOY compared to CON-fed groups. Our last objective was to examine associations between SOY-induced fecal metabolites and previously identified metabolic parameters, such as insulin sensitivity and gene expression patterns in WAT and BAT, which we previously found to be improved in SOY-fed rats. In relation to the first goal, the initial analyses revealed clear fecal metabolome profile separation based on diet consumed and ovarian state. Consumption of the SOY diet by female rats, regardless of ovarian state, resulted in several metabolite changes. Notable ones that were upregulated in these groups include: S-equol; fucose; laminaribose; and several currently uncharacterized metabolites. On the other hand, daidzein; nonadrecylresorcinol; stigmastan-3-ol; hydroxypurine; indole-3-acetic acid; hydrocinnamic acid; and α-tocopherol were reduced in the fecal metabolome profile for these groups. The two pathways likely to be affected in the SOY groups are amino acid (i.e., glycine, serine, and threonine) metabolism and aminoacyl-tRNA biosynthesis. A previous *in vitro* study demonstrated that both genistein and daidzein can activate aminoacyl-tRNA synthetase in osteoblastic cells, resulting in an overall increase in protein synthesis^41^. Interestingly, we found that the weight loss due to SOY associated with preservation of lean mass^12^, which is unlike most weight loss interventions that induce both fat and lean loss.

In both SOY and CON groups, OVX resulted in distinct metabolome changes relative to SHM counterparts, possibly suggesting that endogenous ovarian-derived hormones (e.g., estrogen) might interact with components in both diets to affect the fecal metabolome profiles, although additional evidence in support of this idea is largely lacking. In male cats, sexual maturity and age at time of neutering, i.e. removal of ovarian steroid hormones, can affect circulating metabolites^42^.

Correlation analyses revealed strong associations between previously identified SOY-induced gut microbiota changes^12^ and fecal metabolites. Similarly, others have identified, in soy-fed neonatal White Dutch Landrace pigs, linkages between diet-responsive intestinal metabolites and gut microbes^43^. Importantly, in a human study, the plasma metabolome of vegans living in a Western society and consuming a soy-rich diet differed from that of omnivores, whereas the gut microbial profile between the two groups of individuals was relatively similar^44^. It is not clear the factors that led to differences in circulating metabolites between these two groups of individuals, but suggests that diet may affect microbial metabolism even when microbial shifts are not necessarily present. That study also found that, even though dietary consumption of soy was high, the proportion of vegans able to produce equol was less than those reported in studies examining Asian cohorts^44^. In fact, it is known that there is human genetic heterogenity in equol production following soy isoflavone consumption; this is true, for example, among menopausal women^32^. Only 30–50% of Western individuals produce equol, suggesting that only these individuals experience full metabolic benefits of dietary soy.

In the human gut, daidzein can be metabolized (by gut bacteria) to equol, dihydrodaidzein, and/or O-desmethylangolessin (ODMA). While it appears that several species are involved in daidzein metabolism, the specific species responsible for its metabolism to these various metabolites remain ill-defined^45^. Importantly, the ODMA-producer phenotype (but not the equol-producer phenotype) is associated with obesity^46^. Soy-nut supplementation of adult humans followed by metabolomic analyses reveals three distinct groups: 1) ODMA only producers, 2) equol and ODMA producers, and 3) non-producers of both^47^. Those that produce both ODMA and equol show lower risk for obesity and metabolic disorders but increased pro-inflammatory cytokines. In general, ability to metabolize isoflavones was associated with unique serum and urine metabolome signature profiles^47^. Similarly, soy supplementation to female Bama mini pigs (*Sus scrofa domestica*) resulted in increased expression of lipolytic but decreased expression of lipogenic genes in white adipose tissue^48^.

Given these findings, it is interesting to note that the animals fed SOY experienced significant adiposity reduction compared to CON-fed animals, despite no changes in energy intake or energy expenditure. An intriguing hypothesis is that S-equol, produced by gut microbes, may facilitate weight loss of the host organism via its effect on BAT, a tissue that increases energy expendiure via heat production due to uncoupling of mitochondrial oxidative phosphorylation (i.e., via UCP1 activity). Notably, in the current study, metabolism of daidzein (the precursor of S-equol) is associated with greater *Ucp1* expression in BAT.

Herein, SOY-induced increases in cecal *Prevotella spp*., *Dorea spp*., *Sutterella spp*., and *Phascolarctobacterium spp*. were all positively associated with an increase in fecal S-equol. While most equol-producing bacteria belong to the Coriobacteriaceae or Bifidobacteriaceae family, other bacteria can convert daidzein (and to a lesser extent, genistein) to S-equol^29,34,49–53^. No previous studies have directly shown that *Prevotella spp*. can convert daidzein to S-equol. It is difficult to say definitively whether microbial taxa present in the cecum contribute to the production and excretion of corresponding metabolites. The current studies also only establish correlation not causation. One study in mice showed that dietary supplementation with daidzein and arabinose resulted in lower relative amounts of *Prevotella* but increased the ratio of equol/daidzein compared to supplementation of daidzein alone, suggesting that providing both daidzein and arabinose increases gut bacteria capable of metabolizing daidzein to equol^54^. Comparably, no other studies have shown that the other bacteria listed above can serve as equol producers. Clearly, further work is needed to verify equol-producing capabilities of these bacteria, which can be done by culturing these bacteria in the presence of daidzein or genistein and then measuring potential S-equol production. Such studies could test the *in vitro* ability of these bacteria to metabolize daidzein to S-equol. *In vivo* studies that could be conceived to test this possibility might include feeding specific pathogen-free (SPF) or germ-free mice a SOY-based diet and providing probiotic formulations of each bacteria to determine if such supplementation elevates the amount of intestinal S-equol, especially in germ-free mice that lack a resident gut microbiome. *Bifidobacterium spp*., which can convert daidzein to equol^50^, was surprisingly reduced in the SOY-fed groups. Relative decrease in the amounts of this bacterium was associated with less daidzein in the fecal samples, which might suggest that even the small amounts of *Bifidobacterium spp*. in the SOY group might partially contribute to the conversion of daidzein to S-equol. Repeated sampling of the fecal microbiome and metabolome might also help establish causation in terms of which bacteria are responsible for elevated S-equol present in the SOY-fed groups. To pinpoint how individual components in the SOY diet affect the fecal microbiome and metabolome, individual supplementation of each compound followed by aerobic and anaerobic bacterial cultures and metabolomic analyses of intestinal samples is required.

Importantly, S-equol has been associated, not only with postive metabolic effects, but also with other health benefits, including neuroprotection^31,55–60^. In fact, some human studies have shown that post-menopausal equol producers provided SOY have overall better cognitive performance and improved emotional responses^61,62^. In a cohort of overweight and obese individuals, S-equol production improved cardio-metabolic parameters^63^, similar to those we showed to be improved in the rats fed SOY. S-equol supplementation might also improve menopausal vasomotor symptoms, hot flashes, and osteoporosis^62,64–66^. Anti-cancer properties have also been attributed to this bacterial metabolite^67–73^. Although many positive estrogen-mediated effects are thought to be via estrogen receptor-α (ESR1), the beneficial metabolic effects of S-equol are seemingly independent of ESR1^31^, and are likely due to binding and activating estrogen receptor-β (ESR2)^74–77^. Whether there are sex-differences in the positive metabolic and other effects of S-equol is uncertain, but respresents an important area of future research.

The current studies support a role for S-equol in improving overall metabolic status with greater concentrations of S-equol in SOY-fed rats correlating with reduced adiposity, AT inflammation, and circulating insulin concentrations. Future studies should test the long-term and dose-dependent effects of S-equol in males and females in both intact and gonadectomized states. The results herein provide important physiological parameters and gene expression patterns for such follow-up studies in order to determine the molecular mechanisms driving those protective effects.

Elevated fucose in the SOY groups was also associated with similar positive metabolic AT phenotypes: reductions in weight and inflammatory markers. Elevations in this metabolite might be due to the fact that the SOY diet also had higher oligosaccharide content (Supplementary Table 2). Little is known about the general health effects induced by increased fucose or how fucose affects adipose tissue, but an L-fucose transporter has been identified in relatively high concentrations in adipose tissue^78^. Notably, fucose-binding lectins show anti-lipolytic activity in isolated rat and hamster adipocytes^79^. Further work is needed to determine whether elevated intestinal fucose might mediate some of the beneficial AT effects of a SOY-diet.

It is important to consider potential limitations of the current work. The experimental dietary approach used was one of whole food comparison. That is, our intent was not to determine the effects of isolated phytoestrogens on gut microbial and metabolomic changes, but rather, to determine differences between “soy-based” and “non-soy-based” diets. Indeed, most studies investigating effects of soy have used isolated phytoestrogen supplementation. However, humans consume soy-based diets (e.g., typical Asian diet, vegetarian/vegan diet) whereas others consume more traditional “Western” diets, which tend to be corn-based and provide significantly less whole soy protein. Meanwhile, as is true with many foods in their whole form, the effects of whole food may differ from the effects of the individual dietary constituents. Thus, we sought to first examine the differences between soy-based and non-soy based diets in the most controlled way possible. As such, providing a different protein source in the experimental groups was necessary for this design. However, this approach carries limitations and cannot show direct causative relationships between phytoestrogens and the gut or systemic variables. Future studies should use a more targeted dietary approach in order to identify the active constituents in the experimental soy diet used herein (e.g., individual phytoestrogens, fiber, combinations of specific dietary constituents, etc.).

It is important to note that the oligosaccharide content in the SOY diet was higher compared to the CON diet, as detailed previously (Supplementary Table 2)^12^. Phytoestrogen might interact with other dietary components to affect the fecal metabolome, as shown previously with daidzein and arabinose^54^. To exclude this possibility, future studies will determine whether OVX and SHM rats fed a soy protein isolate diet or supplemented with daidzein (or other single phytoestrogen) show similar gut microbiome and metabolome changes, as identified previously^12^ and in the current studies. As discussed in the original study^12^, cecal weight was significantly greater in the soy group. Since we were not able to normalize this based on dry weight, findings related to the cecal samples should be evaluated in light of this potential limitation. However, importantly, the total fiber content did not differ between the two diets. What varied (in addition to phytoestrogen content), was the oligosaccharide content of the two diets. The soy diet contained ~2% oligosaccharides, whereas the control diet did not; this was done to reflect the known difference in oligosaccharide content between soy and non-soy based diets. Indeed, oligosaccharides are are highly fermentable and considered prebiotic^80^. It is also possible that the soy diet had lower digestibility, providing greater substrate for the gut microbiota to ferment. Future studies should investigate the potential specific role that oligosaccharides may play in mediating gut and systemic metabolic improvements imparted by high soy diets.

In conclusion, the current studies show that OVX and SHM rats fed a SOY-enriched diet possess greater fecal concentrations of S-equol. Ovarian state interacted with both diets to affect other fecal metabolites. S-equol was positively associated with several bacteria previously shown to be upregulated in the SOY group, including *Prevotella spp*., *Dorea spp*., *Sutterella spp*., and *Phascolarctobacterium*. Elevated S-equol in the SOY group was significantly associated with reduced adipose tissue weight, visceral WAT inflammation, and hyperinsulinemia, key biomarkers of metabolic dysfunction. Thus, the current findings support the contention that S-equol is a key mediator between SOY and gut microbiome-positive host health outcomes.

## Methods

### Animals, Physiological, and Gene Expression Assessments

These studies included the same animals and diets as detailed previously^12^. Briefly, the Institutional Animal Care and Use Committee at the University of Missouri, Columbia approved all animal methods that were also performed in accordance with NIH Guidelines for the Care and Use of Laboratory Animals. These studies employed the HCR/LCR rat model^81^. Thirty-five 27-wk-old LCR female rats (generation 32) were either OVX or SHM and *ad libitum* fed either SOY or CON diets for 28 weeks in a 2 × 2 factorial arrangement (n = 7 for OVX CON, 9 for OVX SOY, 10 for SHM CON, and 9 for SHM SOY): (1) OVX/SOY; (2) SHM/SOY; (3) OVX/CON; (4) SHM/CON. SOY diet was formulated to provide ~590 mg/kg diet of soy isoflavones [genistein and daidzein (aglycone equivalents)], whereas CON diet was formulated to exclude any soy isoflavones (Envigo Laboratories Inc., Madison, WI)^12^. Further details on the two diets are listed in Supplementary Table 2. Rats were maintained under controlled humidity and temperature with a 12-h light:12-h dark cycle. Body weight (BW) and food intake were measured weekly. Intraperitoneal glucose tolerance test (IPGTT) was performed 19 weeks post-surgery. At 56 weeks of age, rats were euthanized via CO_2_ inhalation and exsanguination. Individual fat depots, including PGAT, retroperitoneal (RPAT), omental, inguinal SQAT, and interscapular BAT were then dissected and immediately weighed to determine regional fat distribution, histomorphology, and gene expression. Cecal digesta and fresh fecal samples from each animal, who had been previously fasted, were collected at the time of euthanasia, snap frozen in liquid nitrogen, and stored at −80 °C until analysis. The microbiota profiles were determined previously in the cecal digesta^12^. For the current study, 10 ± 0.06 mg fresh fecal sample/animal was extracted at time of sacrifice and all metabolite measurements were normalized physically to this mass. An internal standard was also used to normalize for variation in metabolite recovery and sample preparation. Thus, these results can be considered quantitative. The current studies examined the metabolomics profiles in the fecal samples. Information on these other analyses are detailed in^12^.

#### Metabolomics Analyses

To perform an assessment of how SOY and ovarian state affects gut bacterial metabolite profiles, metabolomics analyses were performed with the fecal samples from all individuals, as detailed previously^82^. To 10 ± 0.06 mg of each fecal sample, 10uL of H_2_O containing 1μg/μL ribitol (internal standard) and 500 μl of 80% methanol were added. This amount of fecal material was extracted for each sample and all metabolite measurements were normalized physically to this mass. An internal standard was also used to normalize for variation in metabolite recovery and sample preparation. Thus, the results can be considered quantitative. The samples were vortexed for 5 seconds, sonicated for 15 min, shaken for 2 hr on an orbital shaker at 140 rpms, and then centrifuged at 13000 g for 15 min. 400 μl of sample volume was collected into a glass vial, dried under a gaseous nitrogen stream, methoximated in pyridine with 40 µL of 15 mg/mL methoxyamine hydrochloride, and then trimethylsilylated with 40 µL MSTFA (N-methyl-N-(trimethyl-silyl)trifluoroacetamide) +1%TMCS (chlorotrimethylsilane) reagent. The derivatized extracts were analyzed as described previously^82^. Metabolic profiling was performed using an Agilent 6890 GC coupled to a 5973 N MSD mass spectrometer with a scan range from m/z 50 to 650 (Agilent Technologies, Inc., Santa Clara, CA). Separation was achieved with a temperature program of 80 °C for 2 min, then ramped at 5 °C/min to 315 °C and held at 315 °C for 12 min, a 60 m DB-5MS column (J&W Scientific, 0.25 mm ID, 0.25 um film thickness) and a constant flow of 1.0 ml/min of helium gas. A standard alkane mix was used for GCMS quality control and retention index calculations. The data were deconvoluted using AMDIS and annotated through mass spectral and retention index matching to an in-house constructed spectra library. The unidentified components were then searched and identified using spectral matching to a commercial NIST17 mass spectral library. The combined identifications were saved as an.ELU file, and the abundance of the ions were extracted using custom MET-IDEA software^83^. The abundances were then normalized to the internal standard, ribitol, and the normalized values were used for statistical comparisons.

#### Statistical Analyses of Metabolomic Data

Multivariate statistical analyses such as PLS-DA, ANOVA, box plots, and volcano plots were performed with the MetaboAnalyst 3.0 program after data pre-treatments, *ie*, normalization to the sum, log transformation and Pareto scaling (http://www.metaboanalyst.ca/). Changes in metabolite abundances were considered statistically significant when their *p* values were less than or equal < to 0.05. Statistical significance of the obtained PLS-DA model was evaluated with a permutation test (permutation number: 1000). The PLS-DA model was considered statistically significant if the permutation test *p* value less than or equal to 0.05. This program was also used to determine based on the overall metabolite changes in the SOY vs. CON groups those pathways that would be predicted to be affected in the SOY groups. The metabolomic data was analyzed by both a one-way ANOVA with Fisher’s least significant difference (using the MetaboAnalyst 3.0 program) and two-way ANOVA using IBM SPSS Statistics Software program (IBM, Armonk, NY).

#### Integrative Correlation Analyses

The cecal microbial community was analyzed as previously described^12^. Multivariate Association with Linear Models (MaAsLin) analysis was performed using default parameters with animal ID being the random effect to assess specific taxa changes (https://huttenhower.sph.harvard.edu/maaslin). These data are included in^12^. The relative abundance of cecal microbial taxa that were significantly upregulated vs. downregulated due to soy consumption were distinguished for correlation analyses. Correlations among the detected fecal metabolites and relative abundances of the cecal microbial taxa were performed using Spearman correlation coefficient using R^84^. Zero filling is common in some metabolomics data processing tools and can skew correlation analyses data. This is often remedied by zero filling with a fixed value or a proportionate noise value; i.e. half the noise. However, our workflow integrates an ion intensity over a defined time window and we seldom get zero values. Thus, zero filling is not a significant issue for our workflow. In reviewing our data, we found 1 unknown metabolite m/z 289.1 (RT 16.0175,Unknown) that had a zero value in three samples. No zero filling, however, was used for this sample. Similarly, correlations among detected fecal metabolites and immune-metabolic/physiological parameters and gene expression data were also examined. Top 25 correlations were identified based on strongest P value association. The usage of ranked-based correlation analyses (i.e. Spearman) addresses potential concern for outliers. Moreover, microbiome/metabolome data can be variable, and thus it is difficult to determine true outliers vs. biologically relevant variations, which can be equally as important. It is for this reason that we used sufficient number of individuals to capture the full range of effects.

## Electronic supplementary material

KEGG permission

Supplementary Informtaion

Supplementary Datset 1

Supplementary Dataset 2

Supplementary Dataset 3

Supplementary Dataset 4

## Data Availability

All data generated from this current study are contained within the manuscript or as supplementary material. Raw and processed metabolomic data are also available at https://sumnerlab.missouri.edu/download/ and within the NIH Metabolomics Workbench database: http://www.metabolomicsworkbench.org/.
