# Supplementary material for: Soy-Induced Fecal Metabolome Changes in Ovariectomized and Intact Female Rats: Relationship with Cardiometabolic Health
Source: Sci Rep. 2018 Nov 15;8:16896. doi: 10.1038/s41598-018-35171-3 (PMC6237990; doi:10.1038/s41598-018-35171-3)
Supplement: Supplementary file 1 — KEGG permission [file 41598_2018_35171_MOESM1_ESM.pdf]

KEGG Database Project
Kanehisa Laboratories
www.kanehisa.jp

Ref: 180293

Permission is granted to Scientific Reports of Springer Nature Ltd to publish both in print and digital under the CC BY 4.0 open access license the following KEGG pathway map image in the article "Soy-Induced Fecal Metabolome Changes in Ovariectomized and Intact Female Rats: Relationship with Cardiometabolic Health" written by Cheryl Rosenfeld and colleagues:

- Glycine, serine and threonine metabolism (map00260)

subject to the condition that the original source is acknowledged by citing at least one KEGG paper.

Permission granted: Miwako Matsumoto

Miwako Matsumoto, Kanehisa Laboratories

Date: 23 October 2018

Copyright holder: Kanehisa Laboratories
