# Supplementary material for: Soy-Induced Fecal Metabolome Changes in Ovariectomized and Intact Female Rats: Relationship with Cardiometabolic Health
Source: Sci Rep. 2018 Nov 15;8:16896. doi: 10.1038/s41598-018-35171-3 (PMC6237990; doi:10.1038/s41598-018-35171-3)
Supplement: Supplementary file 2 — Supplementary Informtaion [file 41598_2018_35171_MOESM2_ESM.pdf]

Supplementary Information

Soy-Induced Fecal Metabolome Changes in Ovariectomized and Intact Female Rats: Relationship with Cardiometabolic Health

Victoria J. Vieira-Potter[1], Tzu-Wen L. Cross[2], Kelly S. Swanson[3,4], Saurav J. Sarma[5,6], Zhentian Lei[5,6,7], Lloyd W. Sumner[5,6,7], Cheryl S. Rosenfeld[7,8,9,10,*]

Affiliations:
[1]Department of Nutrition and Exercise Physiology, University of Missouri, Columbia, MO, 65211, USA

[2]Department of Bacteriology, University of Wisconsin-Madison, Madison, WI 53706, USA

[3]Division of Nutritional Sciences, University of Illinois at Urbana-Champaign, Urbana, IL, 61801, USA.

[4]Department of Animal Sciences, University of Illinois at Urbana-Champaign, Urbana, IL, 61801, USA.

[5]MU Metabolomics Center, University of Missouri, Columbia, MO, 65211 USA

[6]Biochemistry, University of Missouri, Columbia, MO 65211 USA

[7]Bond Life Sciences Center, University of Missouri, Columbia, MO 65211 USA.

[8]Biomedical Sciences, University of Missouri, Columbia, MO 65211 USA

[9]Thompson Center for Autism and Neurobehavioral Disorders, University of Missouri, Columbia, MO 65211 USA

[10]Genetics Area Program, University of Missouri, Columbia, MO 65211 USA

Short Title: Effects of Soy Diet on the Fecal Metabolome

Keywords: Daidzein; Genistein; S-Equol; Metabolomics; Gut Microbiota; Metabolic Disorders; Obesity; Adipose Tissue; Inflammation

***Corresponding author:** rosenfeldc@missouri.edu

Supplementary Figures

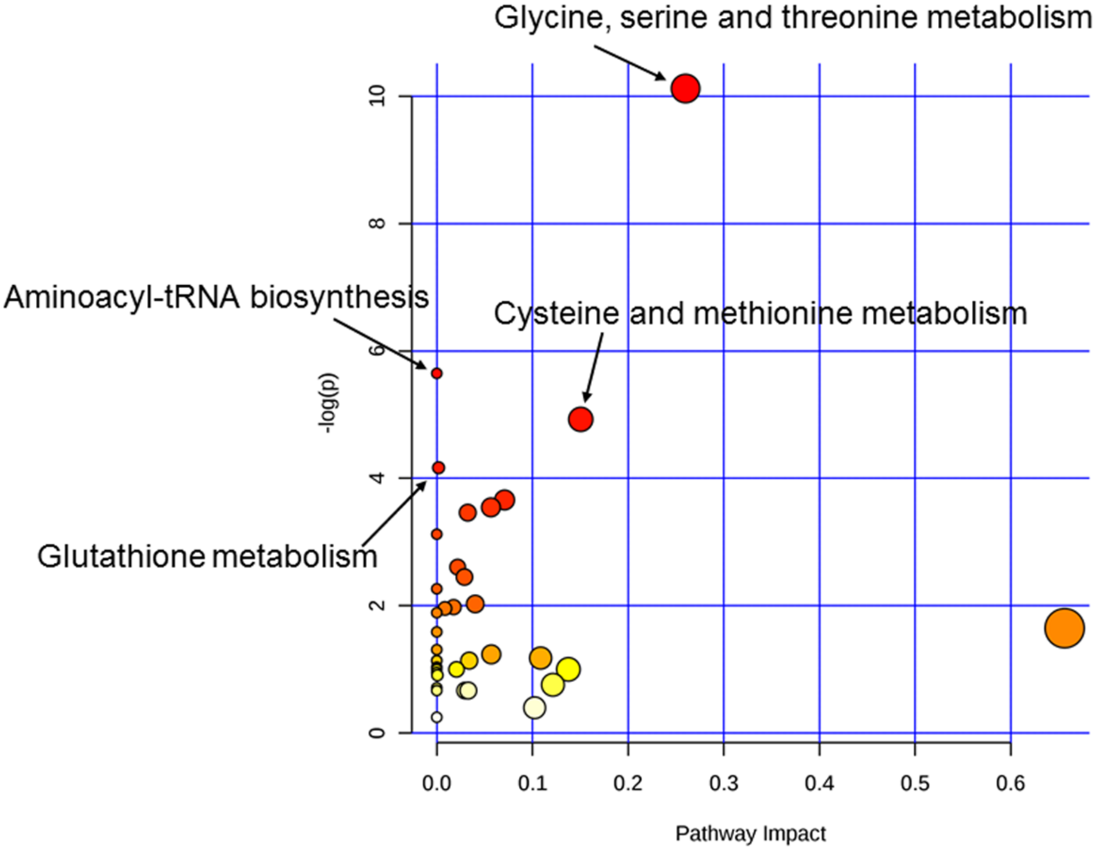

Supplementary Figure 1: Metabolome view of pathway analysis using Metaboanalyst software. For comparison among different pathways, the program calculates the node importance values from centrality measure, which are further normalized by the sum of the importance pathways. The total or maximum importance of each pathway is 1. X- axis shows the impact of the set of significantly different metabolites on a pathway and Y-axis shows the –log(p) values of this correlation between the set of significantly different metabolites and the pathways. The four most significant pathways, as indicated by the lowest *p* values and most hits in the pathway maps, are labeled.

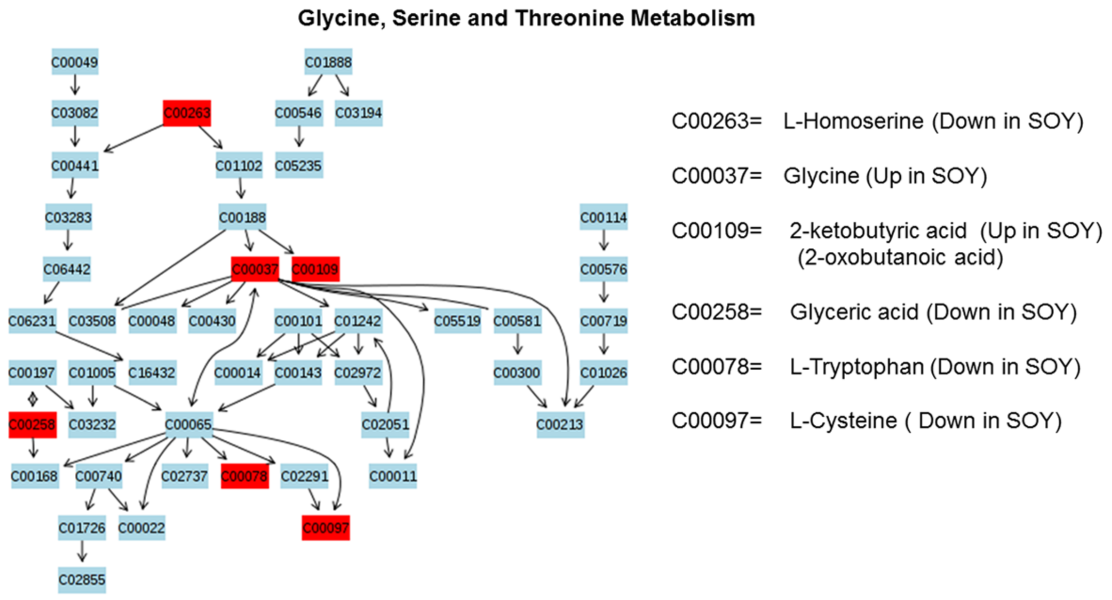

Supplementary Figure 2: Pathway map of glycine, serine, and threonine metabolism, which the most significant pathway associated with SOY-induced metabolite changes, as identified in the pathway analysis in Supplementary Figure 1. The compounds in the pathway map are represented with their KEGG ID. Compounds highlighted in light blue color are not included in the list of significant metabolites, and the ones highlighted in red color are present in the list of significant metabolites.

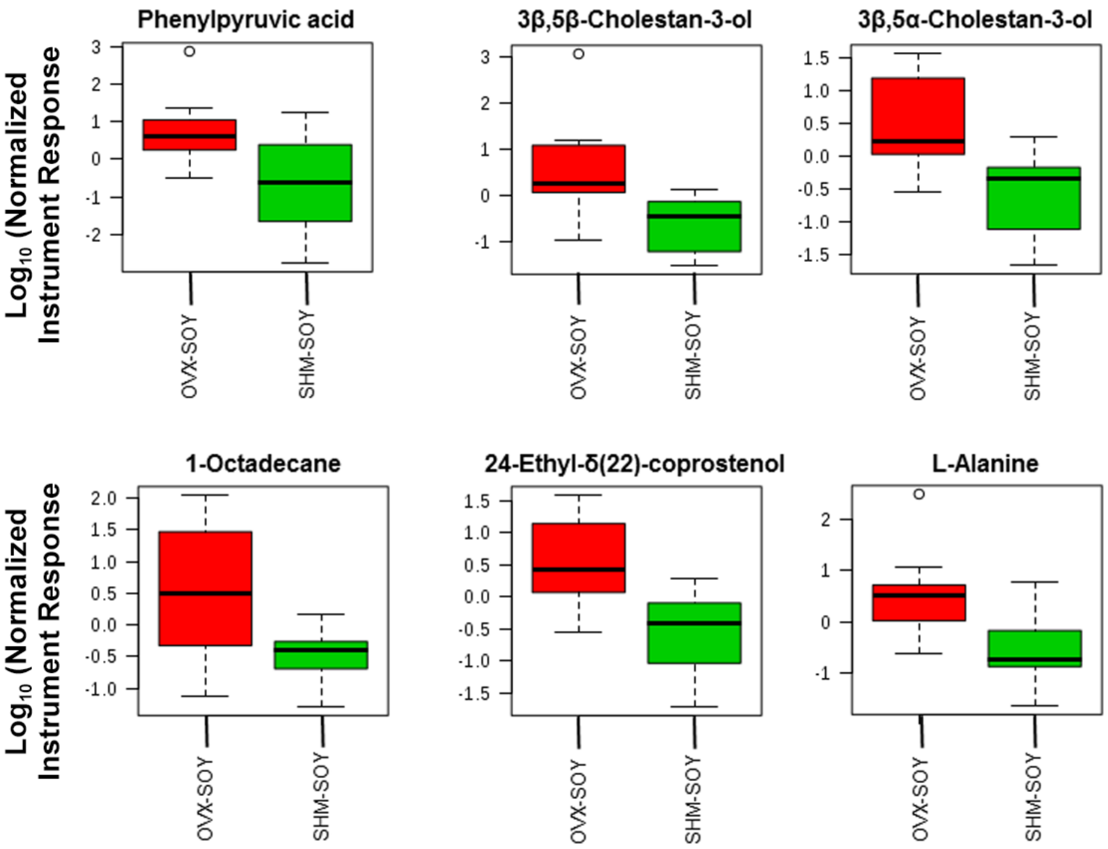

Supplementary Figure 3. Example metabolites that, within the SOY group, were increased in OVX compared to SHM rats. The Y axes are log 10 values of the normalized instrument response for the labeled metabolites (X-axes). The program arbitrarily assigns color codes for the various groups. Phenylpyruvic acid (*p* value = 0.01, *q* value = 0.27). 3β-5β-Cholestan-3-ol (*p* value = 0.01, *q* value = 0.22). 3β,5α-Cholestan-3-ol (*p* value = 0.006, *q* value = 0.18). 1-Octadecane (*p* value = 0.03, *q* value = 0.38). 24-Ethyl-δ(22)-coprostenol (*p* value = 0.005, *q* value = 0.17). L-alanine (*p* value = 0.07, *q* value = 0.46). The complete list of metabolites is provided in Supplemental File 3, and ones that are significantly different are highlighted in orange.

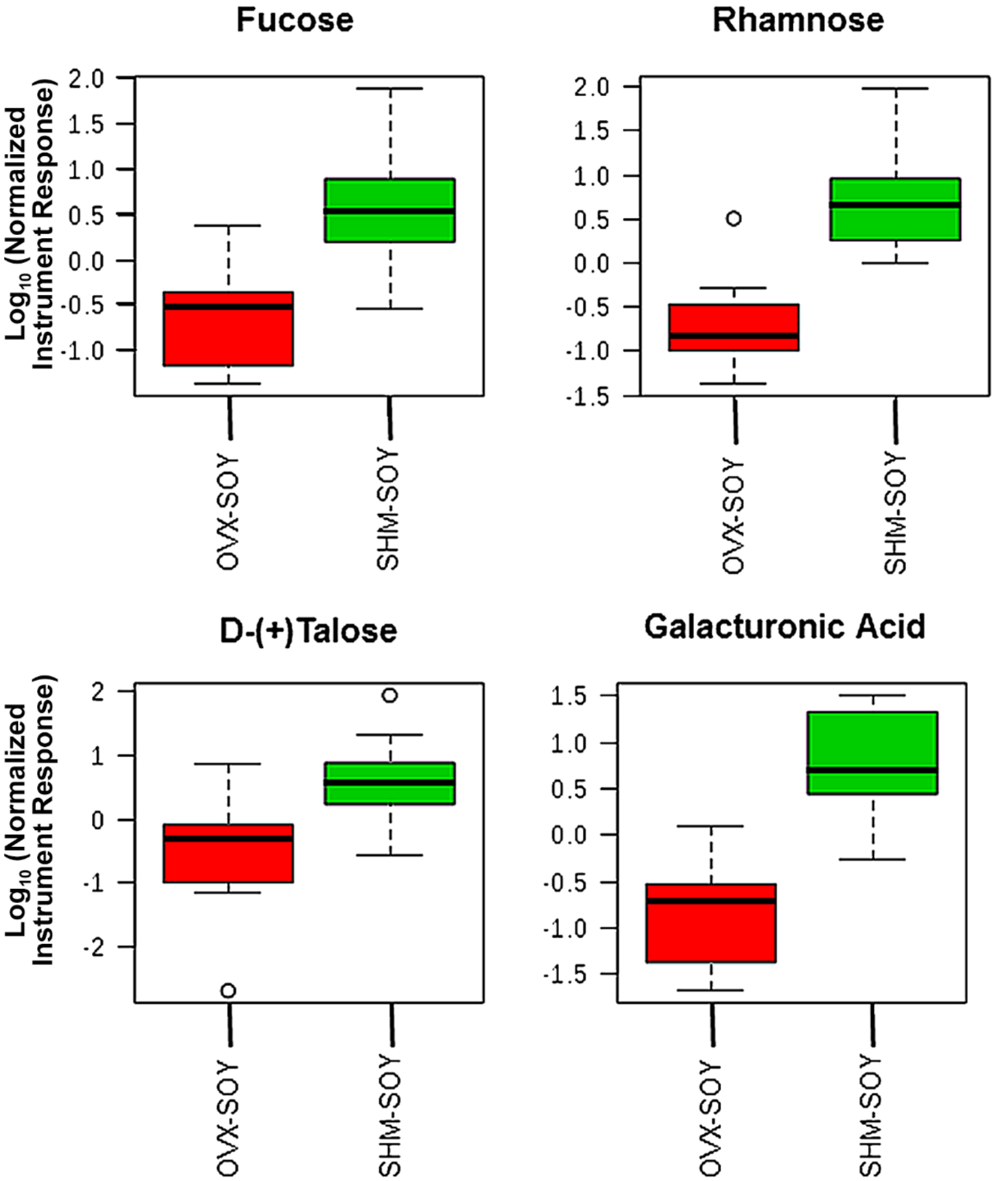

Supplementary Figure 4. Example metabolites that within the SOY group that were decreased in OVX compared to SHM rats. The Y axes are log 10 values of the normalized instrument response for the labeled metabolites (X-axes). The program arbitrarily assigns color codes for the various groups. Fucose (p value = 0.001, q value = 0.11). Rhamnose (p value = 0.0002, q value = 0.03). D-(+)Talose (p value = 0.01, q value = 0.22). Galacturonic acid (p value < 0.0001, q value = 0.008). The complete list of metabolites is provided in Supplemental File 3, and ones that are significantly different are highlighted in orange.

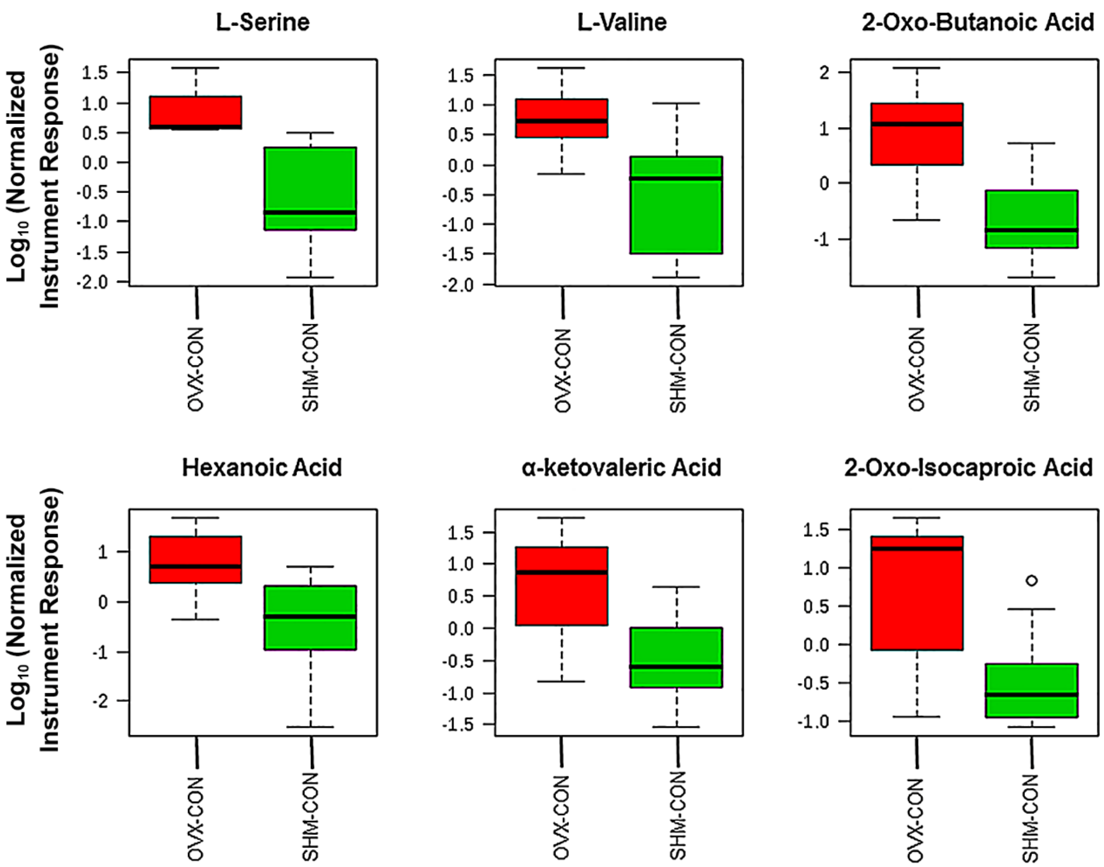

Supplementary Figure 5. Example metabolites that, within the CON group, were increased in OVX compared to SHM rats. The Y axes are log 10 values of the normalized instrument response for the labeled metabolites (X-axes). The program arbitrarily assigns color codes for the various groups. L-serine (*p* value = 0.0001, *q* value = 0.04). L-Valine (*p* value = 0.008, *q* value = 0.33). 2-Oxo-Butanoic Acid (*p* value = 0.004, *q* value = 0.22). Hexanoic acid (*p* value = 0.01, *q* value = 0.36). α-ketovaleric acid (*p* value = 0.02, *q* value = 0.47). 2-Oxo-Isocaproic Acid (*p* value = 0.02, *q* value = 0.47). The complete list of metabolites is provided in Supplemental File 4, and ones that are significantly different are highlighted in orange.

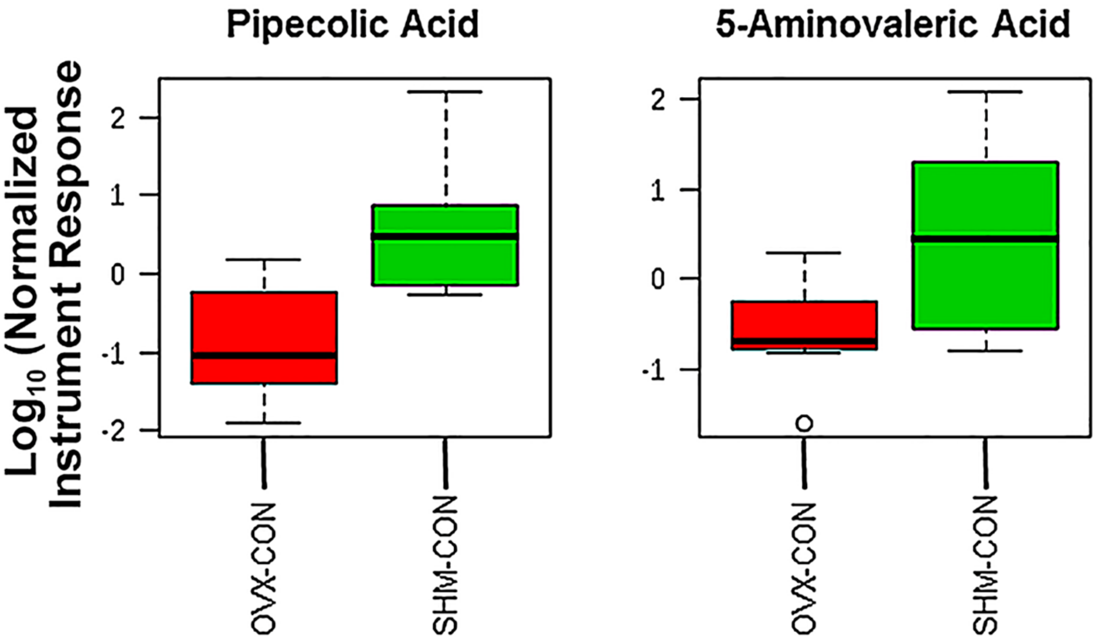

Supplementary Figure 6. Example metabolites that, within the CON group, were decreased in OVX compared to SHM rats. The Y axes are log 10 values of the normalized instrument response for the labeled metabolites (X-axes). The program arbitrarily assigns color codes for the various groups. Pipecolic acid (*p* value = 0.002, *q* value = 0.18). 5-Aminovaleric Acid (*p* value = 0.04, *q* value = 0.47). The complete list of metabolites is provided in Supplemental File 4, and ones that are significantly different are highlighted in orange.

Supplementary Table 1. Two-way ANOVA for main effects of diet and ovarian state, as well as diet by ovarian state interactions for key fecal metabolites that were identified as significant based on confidence testing.

Fecal Metabolite	DIET (SOY vs. CON)	OVARIAN STATE (OVX vs. SHM)	DIET X OVARIAN STATE
**Daidzein**	<0.0001	0.057	0.792
**S-Equol**	<0.0001	0.336	0.386
**Fucose**	<0.0001	0.03	0.018
**Laminaribose**	0.014	0.749	0.554
**5-Nonadecylresorcinol**	0.016	0.732	0.923
**β-Sitosterol**	0.875	0.748	0.347
**3β-Stigmastan-3-ol**	<0.0001	0.774	0.981
**3β,5α-Cholestan-3-ol**	0.882	0.041	0.62
**6-Hydroxypurine**	<0.0001	0.506	0.861
**Hydrocinnamic acid**	0.042	0.213	0.155
**α-Tocopherol**	<0.0001	0.505	0.351
**5-Hydroxy-indole-3- acetic acid**	0.001	0.146	0.484

Table indicates *p* values; significant values are shaded.

Supplementary Table 2. Ingredient and analyzed chemical composition of the phytoestrogen-free (CON) and phytoestrogen-rich (SOY) diets.

	**CON**	**SOY**
**Ingredient**	**g/kg diet (as-fed basis)**	
Corn gluten meal (60% protein)	188	0
Soybean meal (48% protein)	0	260
Corn	388	358
Wheat, soft	231	230
Wheat middlings	73.0	46.0
DL-methionine. FG (99%)	1.0	1.0
L-lysine HCl, FG (78%)	8.0	1.0
Soybean oil	16.0	20.0
Cellulose	58.9	50.6
Mineral mix	5.0	5.0
Calcium phosphate	10.0	8.0
Calcium carbonate	13.0	13.0
Sodium chloride, iodized	2.5	2.5
Magnesium oxide, FG (58%)	0.5	0.5
Vitamin mix	4.0	4.0
Choline chloride, FG (60%)	1.6	0.4
Analyzed chemical composition		
Dry matter (DM, %)	85.2	90.7
	--DM basis--	
Organic matter (%)	95.6	94.2
Ash (%)	4.4	5.8
Crude protein (%)	22.2	20.8
Acid hydrolyzed fat (%)	6.6	6.0
Nitrogen Free Extract (%)	47.6	47.3
**Total dietary fiber (%)**	**19.2**	**20.1**
Insoluble dietary fiber (%)	16.8	19.3
Soluble dietary fiber (%)	2.5	0.8
Gross energy (kcal/g DM)	4.7	4.5
Calculated metabolizable energy (Atwater factor, kcal/g DM)	3.4	3.3
Calculated metabolizable energy (modified Atwater factor, kcal/g DM)	3.0	2.9
**Total phytoestrogen (mg/kg)**	**0**	**585**
**Daidzin/daidzein (mg/kg)**	**0**	**290**
**Genistin/genistein (mg/kg)**	**0**	**190**
**Glycitin/glycitein (mg/kg)**	**0**	**105**
